# Our Contemporaries

**Published:** 1917-09

**Authors:** 


### OUR CONTEMPORARIES

One of our contemporaries devotes the equivalent of two pages of this journal to proving that tuberculous men ought not to be enrolled for military service. Does any sane man doubt it? The only practical point is in regard to healed lesions and incipient cases difficult to detect. A very small minority hold that such cases could endanger others and it is altogether probable that training camps would be a valuable therapeutic aid to the great majority of the tuberculous individuals of this grade. Unfavorable cases should, with ordinary skill and attention, be detected in training camps before harm occurred to either the patients or others.

---

The Atlantic Monthly, June and July, contains articles on Humanism by Paul Shorey, which we recommend to anyone interested in acrometopic English. The style is so different from that of medical writers that we have not been able to comprehend the author's argument but, as a study in words and construction, the articles are well worth reading.

---

M. D., A Journal for Medical Defense, begins its first volume in June. The subscription is $1. Dr. John P. Davin, Editor, 117 W. 76, N. Y. The journal is devoted mainly to counteracting the attempt to socialize the practice of medicine. One of the leading articles is entitled "Is the Practice of Medicine Worth Saving?"

